# Unravelling the Complexities of Bite Force Determinants in Paediatric Patients: A Literature Review

**DOI:** 10.7759/cureus.60630

**Published:** 2024-05-19

**Authors:** Rutuja Patil, Monika Khubchandani, Harikishan Kanani, Ramakrishna Yeluri, Suwarna Dangore-Khasbage

**Affiliations:** 1 Pedodontics and Preventive Dentistry, Sharad Pawar Dental College and Hospital, Datta Meghe Institute of Higher Education and Research, Wardha, IND; 2 Paediatric Dentistry, Sharad Pawar Dental College and Hospital, Datta Meghe Institute of Higher Education and Research, Wardha, IND; 3 Oral Medicine and Radiology, Sharad Pawar Dental College and Hospital, Datta Meghe Institute of Higher Education and Research, Wardha, IND

**Keywords:** paediatric preventive dentistry, muscle of mastication, bite force, review article, primary dentition, maximum voluntary bite force

## Abstract

The amount of maximum voluntary bite force (MVBF) is determined by the combined action of the jaw elevator muscles, which are altered jaw biomechanics and reflex processes. Bite force (BF) measurements can yield valuable information on the activity and function of the jaw muscles. The accuracy of biting force measurements depends on several variables, including age, gender, malocclusion, dental caries, dental prostheses and temporomandibular joint (TMJ). This information is essential for evaluating the development and function of the masticatory system, identifying potential abnormalities or impairments and guiding appropriate treatment interventions for paediatric patients. The aim of this article is to review the literature on the factors affecting bite force and the importance of these factors in assessing dental development and guiding interventions for paediatric patients with bite force-related issues. Additionally, establishing normative values for bite force in different age groups can aid in monitoring growth and detecting any deviations from expected patterns. Measuring bite force in paediatric patients is significant in comprehensive oral health assessment and management.

## Introduction and background

Maximum bite force (MBF) or maximum voluntary bite force (MVBF) is denoted as 'the ability of the mandibular elevator muscles to execute the maximum force of the lower teeth against the upper teeth in favourable circumstances' [1]. Because the jaw elevator muscles are affected by changes in craniofacial biomechanics, one measure of the functional status of the masticatory system is the occlusal bite force (OBF) [2]. Dental professionals employ bite force (BF) analysis extensively to assess mandibular movement, masticatory muscle function and prosthetic device mechanics [3].

Bite force (BF) measurements in children and adolescents varied from 246.22 to 489.35 N and from 5.69 to 16.1 kg [4]. Furthermore, the range of average biting force values for different devices, such as strain gauge transducers, biting forks, pressure-sensitive sheets, quartz and foil transducers, pressurised rubber tubes, gnathodynamometers and force sensing resistors, is from 226.52 to 428.08 N. These results shed light on the variety of biting force exertions seen in paediatric populations and the effectiveness of various devices in modifying or sustaining bite force levels [4].

Much work has been done to analyse the relationships between bite force and a variety of different variables; some examples of the factors that can be examined include craniofacial dimensions, head posture, chewing performance, clenching strength, masticatory muscle thickness and occlusal contacts. The measurements of bite force are susceptible to fluctuations in the methods used for the experiments, the design of the instruments, the researcher's attitude and approach and the child's cooperation [5]. Bite force values can be acquired from an individual directly or indirectly. Electromyographic (EMG) activity, MVBF measurement and masticatory efficiency are a few of the criteria employed for BF assessment by clinical methods. Masticatory performance and EMG, on the other hand, cannot be measured as numerical data, in contrast to MVBF [6].

This review article gives information about the special anatomical, developmental and behavioural elements that affect biting force in children. This entails investigating how biting force dynamics are shaped by dental occlusion, craniofacial growth patterns and muscle development. The study aims to give insights into paediatric dentistry, orthodontics and child development by synthesising existing literature, which will eventually help in better understanding and managing biting force-related difficulties in young patients.

## Review

Age

Biting force is strongly impacted by age, as it displays the variability in the masticatory system's performance during the course of a person's life. An elderly person's biting force may decrease due to several causes, such as diminished bone density, tooth loss and muscular weakness. In younger people, biting force frequently rises with age as the jaw muscles and craniofacial anatomy mature. A significant relationship is observed between the force of biting and the age of children with permanent teeth, specifically between six and 18 [7]. During the course of mammalian ontogeny, the masticatory system goes through significant alterations as the teeth are shed and replaced. The shape of the dental row also adjusts to accommodate the growth and emergence of new teeth. The development of the masticatory muscles leads to the assumption that bite forces increase as ontogeny progresses [7].

Olthoff et al. (2007) conducted a study on the OBF in individuals ages eight and 68 years. They exhibited an occlusal bite force that increased steadily from youth onwards, became constant between 20 years and 40 years and then declined as the normal ageing process caused the loss of muscle force. Basically, occlusal bite force correlates with age and with the different phases of life. Comprehending the age-related fluctuations in OBF might affect several physiological functions [8].

**Figure 1:**
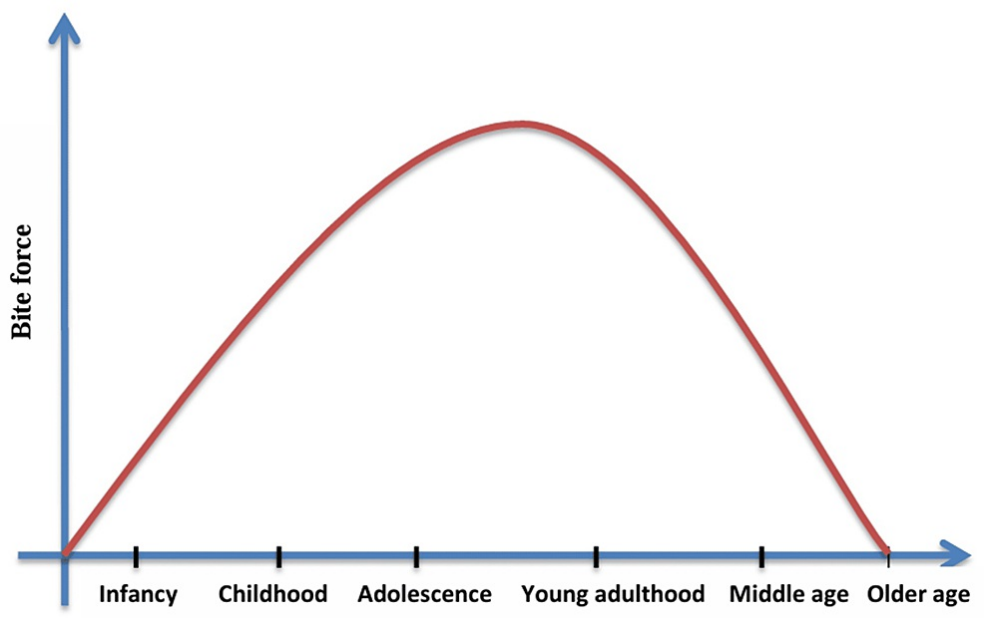
Graph depicting bite force versus age

The maximum voluntary bite force (MVBF) shows a propensity to rise with age, increasing at about 17 years for females and 20 years for males, according to Kovero et al. (2002), who examined the maximum occlusal bite force (MOBF) in various age groups. This suggests that depending on their gender, different people experience age-related changes (Figure 1) [9].

Gender

Variations in craniofacial structures, muscle mass and hormones lead to changes in biting force. Research has shown that females tend to bite less forcefully than males. A study by Pizolato et al. (2007) [10] found that the size of type IIa/fast-twitch oxidative fibres found in males' masseter muscles is bigger than that found in females. Because of these fibres, bite force increases as they show higher activity of myosin ATPase, an enzyme that catalyses the hydrolysis of adenosine triphosphate (ATP), which releases the energy required for muscle contraction. These fibres contract more rapidly than other fibres, and they are designed for an anaerobic metabolism, allowing them to generate power quickly without the need for oxygen. The difference in hormone levels such as thyroid hormone, oestrogen and testosterone might be the reason behind the difference in facial muscle fibre composition [10,11]. The same was demonstrated by a study conducted by Nandini et al. (2022) that measured mastication forces in young children aged 4-6 years. During the growing age of children, females displayed reduced biting force compared to males, which clearly implies that there are physical differences in chewing behaviour between males and females. This difference may include reduced teeth size and weaker muscles in females compared to males [3].

**Figure 2:**
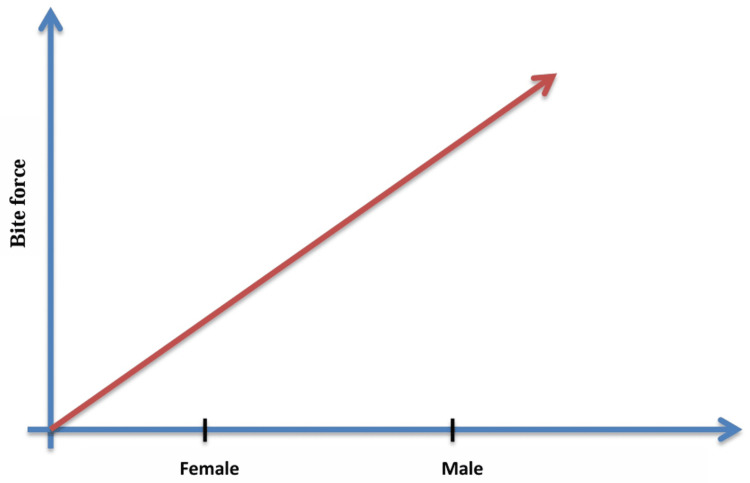
Graph depicting bite force versus gender

Palinkas et al. (2010) conducted a study on individuals from various age groups, ranging from seven to 80 years old. They found a notable difference between males and females in terms of mean, with males having a 30% higher mean compared to females. So, it is important to consider gender when measuring bite force [12]. However, Serra et al. (2007) observed that regardless of gender, the maximum voluntary bite force measurements are the same [13]. Ferrario et al. (2004) addressed the males having higher biting force by bringing attention to their larger teeth, which are surrounded by a larger periodontal ligament area. Periodontal ligaments distribute tension when chewing in addition to connecting the teeth to the surrounding bone. Because their larger periodontal ligament areas give them greater leverage and support from the surrounding tissues, males may bite harder [14]. This study suggests that physiological variations in tooth size and periodontal ligament area may affect biting force between genders and may also affect the temporomandibular joint (TMJ) (Figure 2).

Craniofacial morphology

Maximum bite force is related to the skeletal measurements of the craniofacial morphology, such as gonial angle, mandibular inclination and the ratio of anterior to posterior face height. Elevator muscles show a stronger mechanical advantage when the gonial angle is acute and the ramus is more vertical [2].

**Figure 3:**
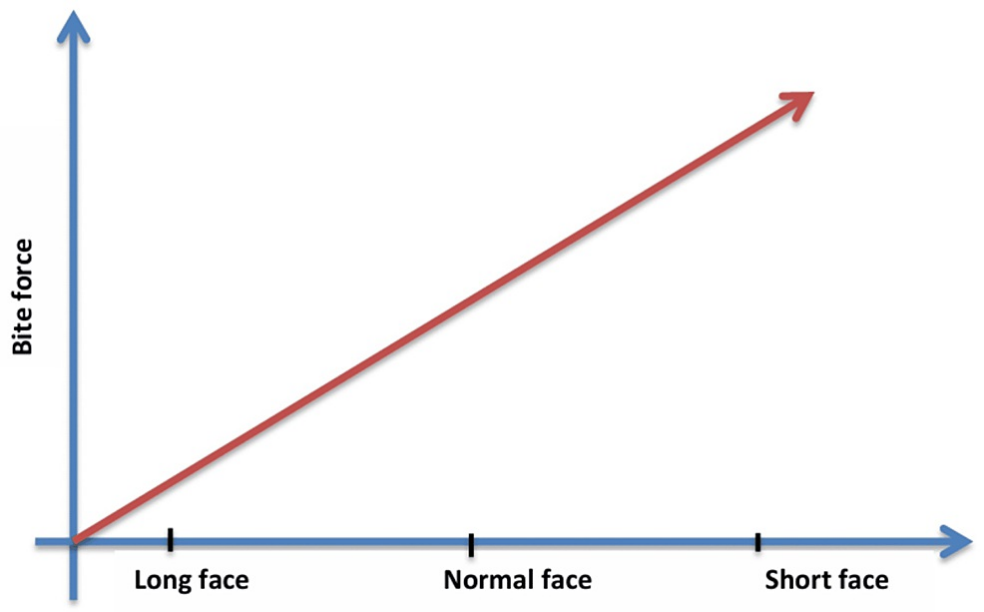
Graph depicting craniofacial morphology versus bite force

According to Proffit and Fields (1983), in those with normal facial shape (Jarabak ratio or facial height ratio {FHR}: 8), the ratio of posterior facial height (sella {S}-gonion {Go}) to anterior facial height had an average occlusal bite force (OBF) in the molar area that was twice as high as people with long face types [15]. Furthermore, compared to people with normal facial height, those with short face structures generated considerably larger forces [16]. The maximum occlusal bite force (MOBF) was determined to be highest in people with short facial structures and lowest in those with long face types, as reported by Abu-Alhaija et al. (2018) (Figure 3) [17].

Malocclusion

The word 'malocclusion' refers to the misalignment or incorrect placement of the jaws and teeth, and it implies that this might negatively impact the masticatory system's capacity to break down and process food, which decreases the bite force value significantly [18].

**Figure 4:**
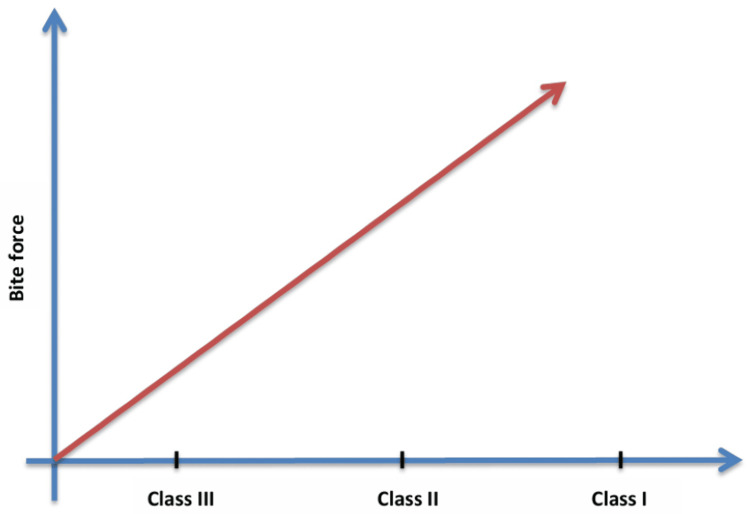
Graph depicting malocclusion versus bite force

In a study conducted by Devi et al. (2022), only instances categorised as Angle's class II malocclusion (dental malocclusion) showed a lower occlusal contact area and correspondingly decreased biting force [19]. Henrikson et al. (1998) compared the masticatory abilities of females with class II malocclusion with those having class I occlusion, between ages 11 and 15. They found that compared to females with class I occlusion, females with class II malocclusion show poor masticatory function [20]. Teenage females can have a negative impact on chewing food if their teeth are not aligned properly, especially in class II malocclusion. Understanding these differences may be critical to the treatment's success since maximum voluntary bite force is an important consideration in orthodontic treatment planning and the masticatory function of the patient (Figure 4).

Temporomandibular joint

'Temporomandibular disorders' (TMD) include pain-related symptoms and indicators in addition to anatomical and functional abnormalities of the masticatory system, with a focus on the temporomandibular joints (TMJ) and the masticatory muscles [21]. In order to discover the precise reasons for TMD in each case, dentists must do a comprehensive examination and evaluation. This is because different treatment strategies may be necessary depending on the underlying causes of TMD, as it affects the bite force measures in patients. The major causes of temporomandibular joint pain are malocclusion, bruxism, trauma, muscle tension, anxiety or stress, developmental factors, genetic predisposition, orthodontic treatment, etc.

**Figure 5:**
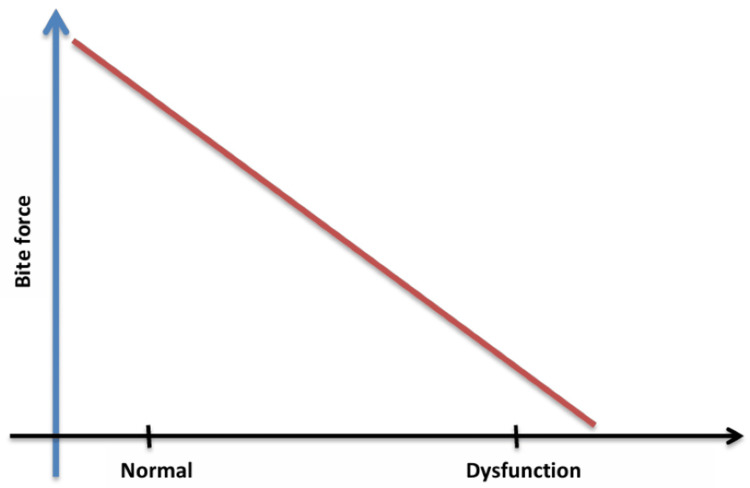
Graph depicting temporomandibular joint versus bite force

Pereira-Cenci et al. (2007) conducted a study on maximum bite force and its association with the temporomandibular joint, and they inferred that there was no discernible effect of temporomandibular disorders (TMD) on biting force. In male TMD subjects, maximum bite force (MBF) was found to be correlated with their weight and height. Again, in their study, they concluded that TMD signs and symptoms were linked to an increase in long face and overjet features, but no one feature was able to accurately predict dysfunction. As there was no noteworthy finding, it would not seem to be feasible to draw any clear conclusions regarding the existence of any specific morphology in teenagers exhibiting signs or symptoms of TMJ dysfunction [7]. While many authors believe that the muscle pain in the TMD with biting decreases the maximum voluntary bite force, Kogawa et al. (2006) reported that TMJ discomfort was the most common reason for the limited biting force (Figure 5) [22].

Molar relationship

The role of the molar relationship on bite force was studied by Nandini et al. (2022) among 4-6-year-old children. They concluded that the flush terminal plane molar relationship showed the highest bite force compared to the mesial and distal step molar relationships [3]. Koolstra et al. (1988) stated the same fact in relation to the mandible; the muscle components' courses of action, such as contraction and relaxation, may vary. Thus, the increased occlusal bite force may be caused by improved masticatory muscle function, as edge-to-edge molar relations give maximum occlusal contact area in the flush terminal plane [23]. It is a dynamic process that leads from deciduous to early-mixed, late-mixed and permanent dentitions. As a result, the biting force applied at a certain time may vary depending on the kind of dentition [18].

**Figure 6:**
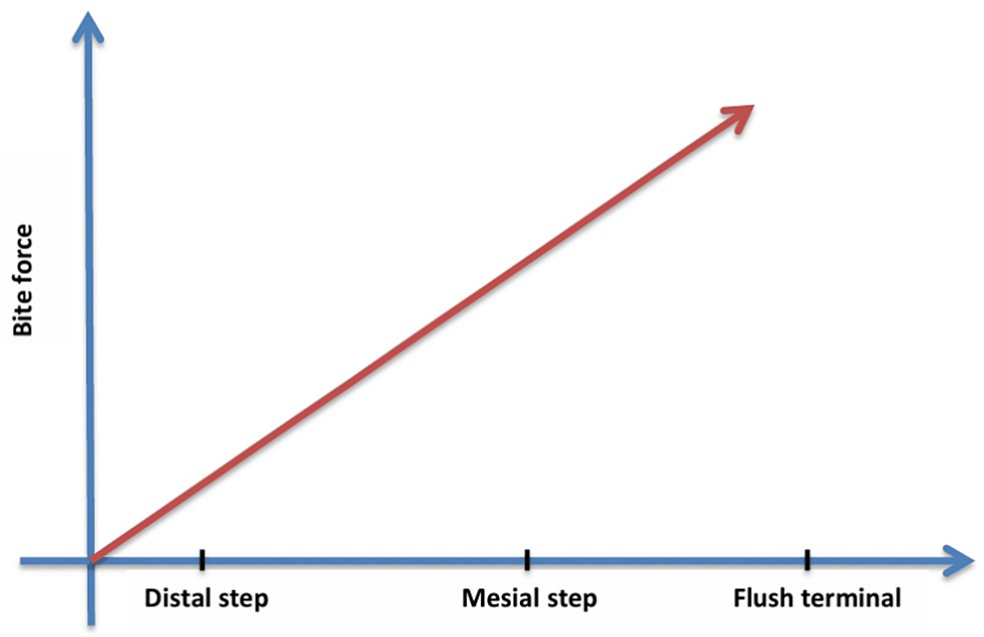
Graph depicting molar relationship versus bite force

Singh et al. (2012) concluded that the intercuspal bite is considerably larger given the larger biting force, whereas the interincisal bite decreases as the mandible moves towards the anterior of the mouth and forms an edge-to-edge relationship. This shows that when an individual bites, force is distributed across the dental arch, with more force in the posterior region [24]. In the research of Sonnesen et al. (2001), a considerably lower biting force was seen in the unilateral crossbite group as compared to the control group due to changes in the muscle function linked to the unilateral crossbite, and this difference persisted as the crossbite group aged and developed [25]. According to these results, it is best to correct unilateral posterior crossbite as soon as possible in order to maximise function (Figure 6).

Jaw elevator muscles

During maximum clenching, the muscles responsible for raising the jaw exert their utmost voluntary power. Bite force levels peak at 15-20 mm of interincisal distance and increase as clenching is performed with a larger degree of jaw opening. This probably corresponds to the ideal sarcomere length in the jaw elevator muscles. But after that, the biting force decreases as the jaw opens more. The length-tension connection is a characteristic that must be taken into account when determining the maximum bite force using devices that adjust bite height and jaw separation [2].

Dental status, prostheses and implants

For patient satisfaction, long-term oral health and the functional outcome of the rehabilitation, there are some factors that are important to consider, such as prosthesis type and location, particularly implant-supported restorations, as they impact the bite force distribution. The positive correlation between the position of the teeth and the number of teeth present, as well as maximum and submaximal biting force, is found in a research study conducted by Lassila et al. (1985) who concluded that people with more balanced dentition and a maximum number of teeth bite harder and have the maximum voluntary bite force value. This association highlights the importance of occlusal stability in optimising biting force capacity [26].

Hidaka et al.'s (1999) research highlighted the association between the contact point of the teeth and the number of teeth present in establishing the maximum voluntary bite force. Particularly in the posterior dental arch, there is a maximum bite force, which is in direct connection with maximum tooth contacts. Bite force increases because of the increased surface area of the occlusal contact in the posterior teeth, which sustains force during masticatory activity. For example, if occlusal contact regions double, the maximum bite force level rises from 30% to 100% indicating that occlusal contact distribution has a major impact on the development of biting force [27].

In the research conducted by Kampe et al. (1987), they examined maximal biting force measurements in individuals with and without dental fillings, concentrating on both the incisor and molar teeth. As a result, the people without filling, especially in the incisor area, bite harder than the people with filling [28]. This implies that dental procedures such as fillings have an impact on an individual's occlusal function since they might change the dynamics and distribution of biting force. Miyaura et al. (2000) examined maximum bite force values for complete dentures, fixed partial dentures, detachable partial dentures and entire natural dentition groups in relation to various dental diseases. They discovered that those with natural dentition had the highest bite forces; biting forces compared to the natural dentition group were 80%, 35% and 11% for the fixed partial denture, detachable partial denture and complete denture groups, respectively [29]. This implies that different dental prostheses have lower biting force capacities than the natural dentition [29].

Dental prostheses, implants and biting force are significantly correlated when assessing functional outcomes and treatment effectiveness. Bite force may be strengthened since well-fitting implants and other well-fitting prostheses stabilise and re-establish the occlusal functioning, while bite force may decrease because of ill-fitting prosthetics or ill-fitting implants [29]. The early exfoliation of the primary tooth may affect the distribution of biting force. As a result, the normal alignment and occlusal contacts of the growing dentition may be altered. However, this phenomenon may influence occlusal stability and chewing efficacy over the development into adolescence [4].

**Figure 7:**
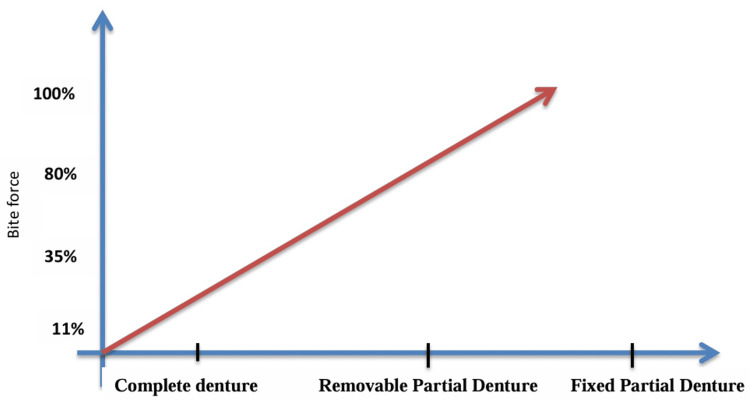
Graph depicting bite force versus prosthesis

According to Aishwarya et al.'s (2021) study, biting force 'should also be factored while accounting for the effect of stainless steel crowns (SSCs) on occlusion'. Stainless steel crown (SSC)-induced occlusal alterations were temporary and gradually diminished over time. To support this result, the prematurities were found lower from baseline to the fourth week. Occlusal changes after placing a stainless steel crown (SSC) are temporary; informing the patient and thoroughly evaluating the long-term stability are important (Figure 7) [30].

Opening wide of the mouth

According to a prior study by Lindauer et al. (1993), the masseter muscle's electromyographic activity decreased as the jaw opened, but biting force values remained constant. Similarly, maximum bite force magnitudes between 15 and 20 mm anterior vertical jaw opening have been obtained when masseter muscle activity levels were maintained constant [31].

It is shown in a different study by Mackenna and Türker (1983) among 16 subjects ages between 22 and 32 years that the greatest incising force peaks at around 17 mm of incisal opening [32]. According to the study, there is an ideal point beyond which there is a drop in the maximal incising force strength due to both decreasing and rising jaw spacing. This result suggests a key range where biting power decreases for the ideal incisal opening. Therefore, the relationship can be used to design dental prostheses and rehabilitation techniques that will ensure the optimal function and comfort in the incising duty [32].

**Figure 8:**
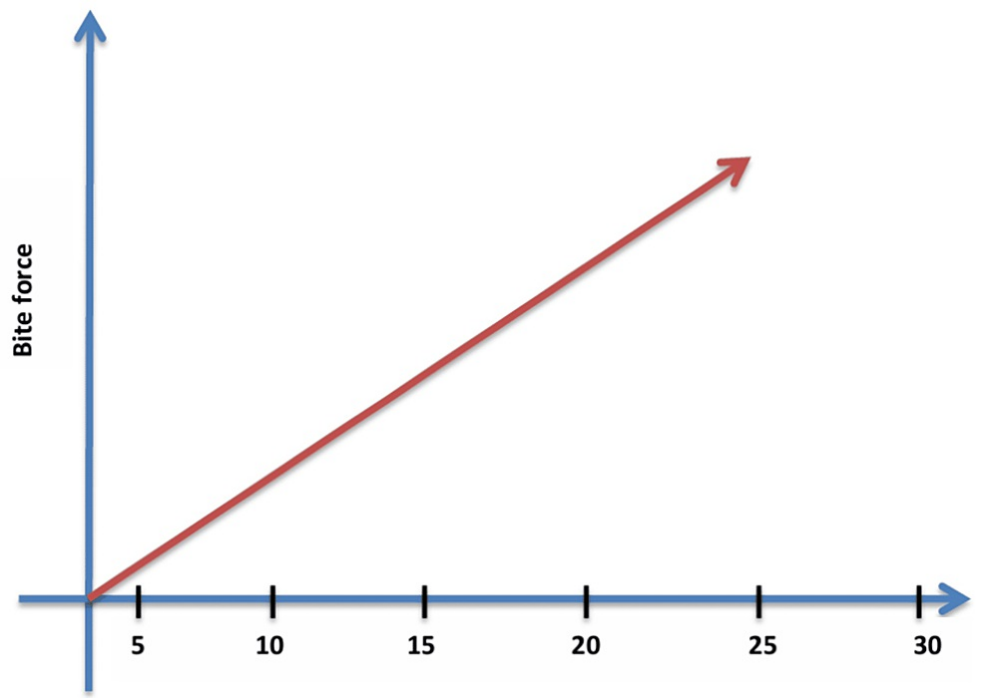
Graph depicting mouth opening in millimetres versus bite force

According to Manns et al. (1979), between 15 and 20 mm at jaw apertures, anterior activity levels indicate little muscle activation. It is interesting to note, however, that at these apertures, the activity is similar to what was achieved at the molar region at 9-11 mm during the experiment [33]. These results demonstrate the need to understand muscle activity patterns that will contribute to consistent muscular behaviour across a jaw position and to effective bite force, other dental procedures and rehabilitation planning (Figure 8).

Head posture

Hellsing and Hagberg's (1990) cephalometric measurements demonstrated that the altered head position altered the positioning of the hyoid bone relative to the mandible and pharyngeal airway. The changes in the interaction of determining the muscle groups of the depressors and the elevators may be the cause of the cephalometric variations in the hyoid bone [34]. This possibility was one of the factors that might have aided in producing the most potent biting force ever recorded.

Unilateral and bilateral measurements

Therefore, assessing correlations between biting force measurements taken unilaterally and bilaterally is necessary to determine the overall functioning of the masticatory system. Unilateral measurements assess the force on one side of the jaw and help identify differences and specific problems. Bilateral measurements help determine the stability of the bite and check adequacy in chewing. Both unilateral and bilateral assessments can be done to correlate and to determine the extent of differentiation to ensure that the appropriate treatment regimen is established to enhance the even distribution of the pulling capabilities.

van der Bilt et al. (2008) published a study about significantly higher muscle activities in the right and left masseters and the anterior temporal muscles during bilateral clenching compared to unilateral clenching [35]. During unilateral clenching, the ipsilateral and the contralateral side of the masseters perform the same activity. During bilateral clenching, the stress on the masseters and temporal muscle differs. The anterior temporal muscle activity on the ipsilateral side is still more significant than on the contralateral side. So, it is evident that during clenching, unilateral and bilateral clenching makes different stresses on the jaw [35].

**Figure 9:**
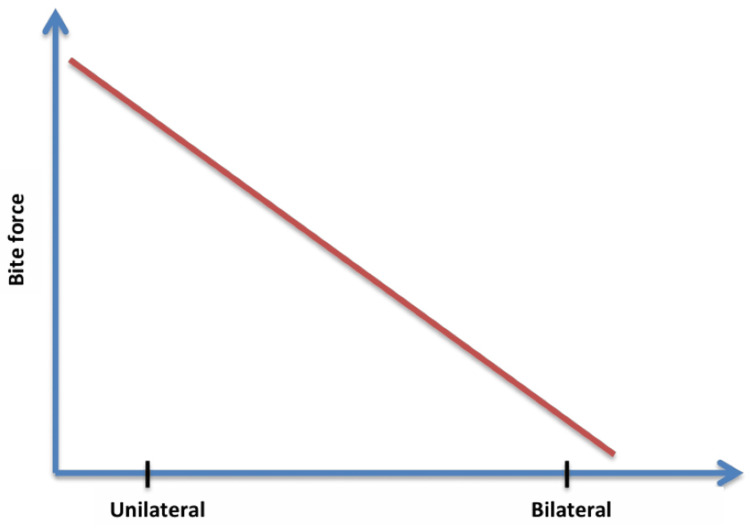
Graph depicting measurements versus bite force

Shinogaya et al. (2001) conducted a study, which proved that bilateral clenching is a more strong bite force than unilateral clenches [36]. According to van Eijden (2000), the force applied at the joint on the balancing side is often higher than that on the working side during unilateral clenching, which is a highly asymmetric movement [37]. When performing unilateral activities, the functional distinctions between the two sides of the jaw are reflected in this imbalance in force distribution. Comprehending this asymmetry is essential for evaluating bite force and jaw function and identifying issues associated with temporomandibular joint diseases or imbalances in the muscles (Figure 9).

Position of recording device in dental arch

Tortopidis et al.'s (1998) research indicates that when maximal biting force is recorded just on one side between the first molar and second premolar teeth, it shows the least amount of fluctuation. No matter where in the dental arch it is located, maximal biting force shows very little within-subject variation when measured at various times [38].

Significantly, the transducer's location during the measurement is important; larger maximum forces are achieved when the biting force is captured more posteriorly. These observations highlight how crucial it is to standardise measuring procedures and take anatomical placement into account in order to accurately estimate biting force in clinical and research contexts [38].

## Conclusions

This literature analysis concludes by highlighting the complex relationship between the many variables determining biting force in paediatric patients. Bite force dynamics during childhood are greatly influenced by a number of variables, including dental occlusion and alignment, craniofacial growth and development, muscle development and coordination. Clinicians in orthodontics and paediatric dentistry must comprehend these elements in order to correctly diagnose biting force-related problems and develop effective treatment plans. To fully understand how these variables interact and what effect they will have in the long run on children's dental health and general development, more study is necessary. In the end, physicians may work to optimise oral function and support healthy development in paediatric patients by addressing these aspects in their entirety.
